# The biology of interleukin-6 family cytokines is regulated by glycosylation

**DOI:** 10.1042/BCJ20240769

**Published:** 2025-05-14

**Authors:** Lisa Kohrs, Falk F. R. Buettner, Juliane Lokau, Christoph Garbers

**Affiliations:** 1 Institute of Clinical Biochemistry, Hannover Medical School, 30625, Hannover, Germany; 2 Proteomics, Institute of Theoretical Medicine, Faculty of Medicine, University of Augsburg, 86159, Augsburg, Germany

**Keywords:** glycosylation, gp130, interleukin-6, interleukin-6 receptor, signal transduction

## Abstract

Cytokines of the interleukin-6 (IL-6) family are important soluble mediators with crucial roles in developmental processes, tissue homeostasis, regeneration, and immune cell differentiation. Overshooting activities of IL-6 and other cytokines are found in all inflammatory diseases, making them attractive therapeutic targets for the treatment of patients with rheumatoid arthritis or inflammatory bowel disease. Multiple mechanisms exist that control cytokine activity and prevent excessive cytokine signaling under normal conditions. In this review, we summarize how the biology of IL-6 family cytokines is regulated by glycosylation, a process in which carbohydrate chains are covalently linked to protein molecules. The attached carbohydrates, which are generated and modified by enzymes located in the endoplasmic reticulum and/or the Golgi apparatus, can display huge structural diversity and are linked either via asparagine (*N*-glycans), serine and threonine (*O*-glycans), or tryptophan residues (*C*-glycans). We describe how glycosylation affects synthesis, receptor binding, signaling and plasma half-life of the cytokines and protein stability, transport to the cell surface, ligand binding, proteolysis, internalization, and recycling of their receptors. Finally, we discuss how knowledge about glycosylation can be used for the design of novel therapeutics targeting IL-6 family cytokines or their receptors.

## Introduction

Cytokines are a heterogeneous group of secreted glycoproteins that have crucial roles in practically all physiological processes, including regeneration, development, tissue homeostasis, and immune cell differentiation. Already in 1986, a cDNA coding for B cell stimulatory factor 2 was cloned [1], which was later renamed and is nowadays known as interleukin-6 (IL-6) [2,3]. IL-6 is part of a large family of cytokines, whose members have, despite several similarities in terms of protein structure, use of signal-transducing receptors and activation of similar signaling pathways in their target cells, unique functions in health and disease [4–6].

IL-6 is largely absent in healthy individuals, but its serum level can rise dramatically in patients with inflammatory diseases. The involvement of IL-6 in basically all inflammatory diseases makes it an attractive therapeutic target. Indeed, the first antibodies against IL-6 and the IL-6 receptor have been developed more than 20 years ago and are in clinical use for patients with rheumatoid arthritis [7,8]. Multiple mechanisms exist that control cytokine activity and prevent excessive cytokine signaling under physiological conditions.

**Figure 1:**
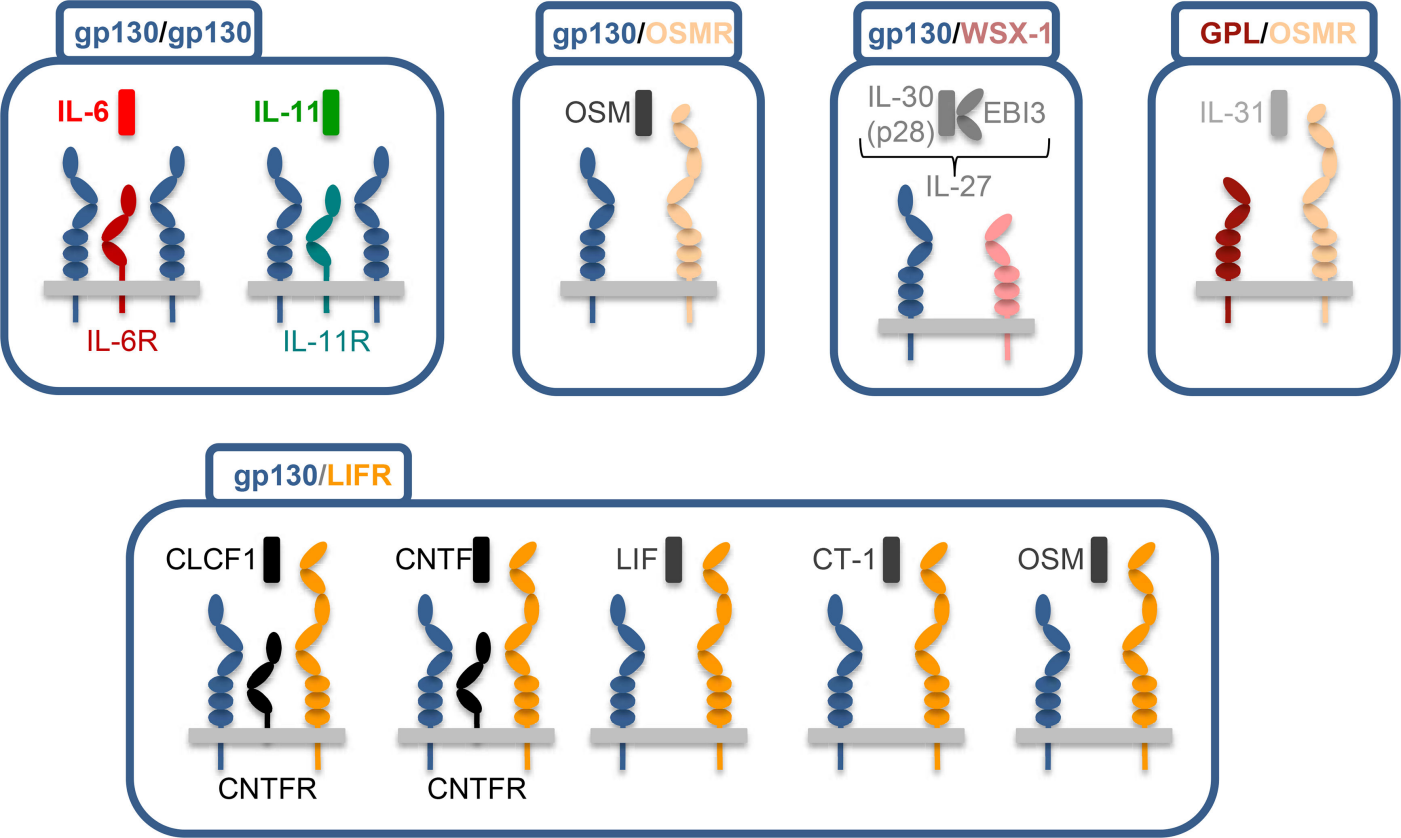
The interleukin-6 family of cytokines. Schematic overview of the IL-6 family of cytokines. Each individual cytokine is shown above the two signal-transducing β-receptors it uses to activate intracellular signaling cascades. IL-6, IL-11, CLCF1, and CNTF require additional non-signaling α-receptors for formation of the signal transduction complex, which are also shown. Cytokines are grouped according to the β-receptor combination they use.

**Figure 2:**
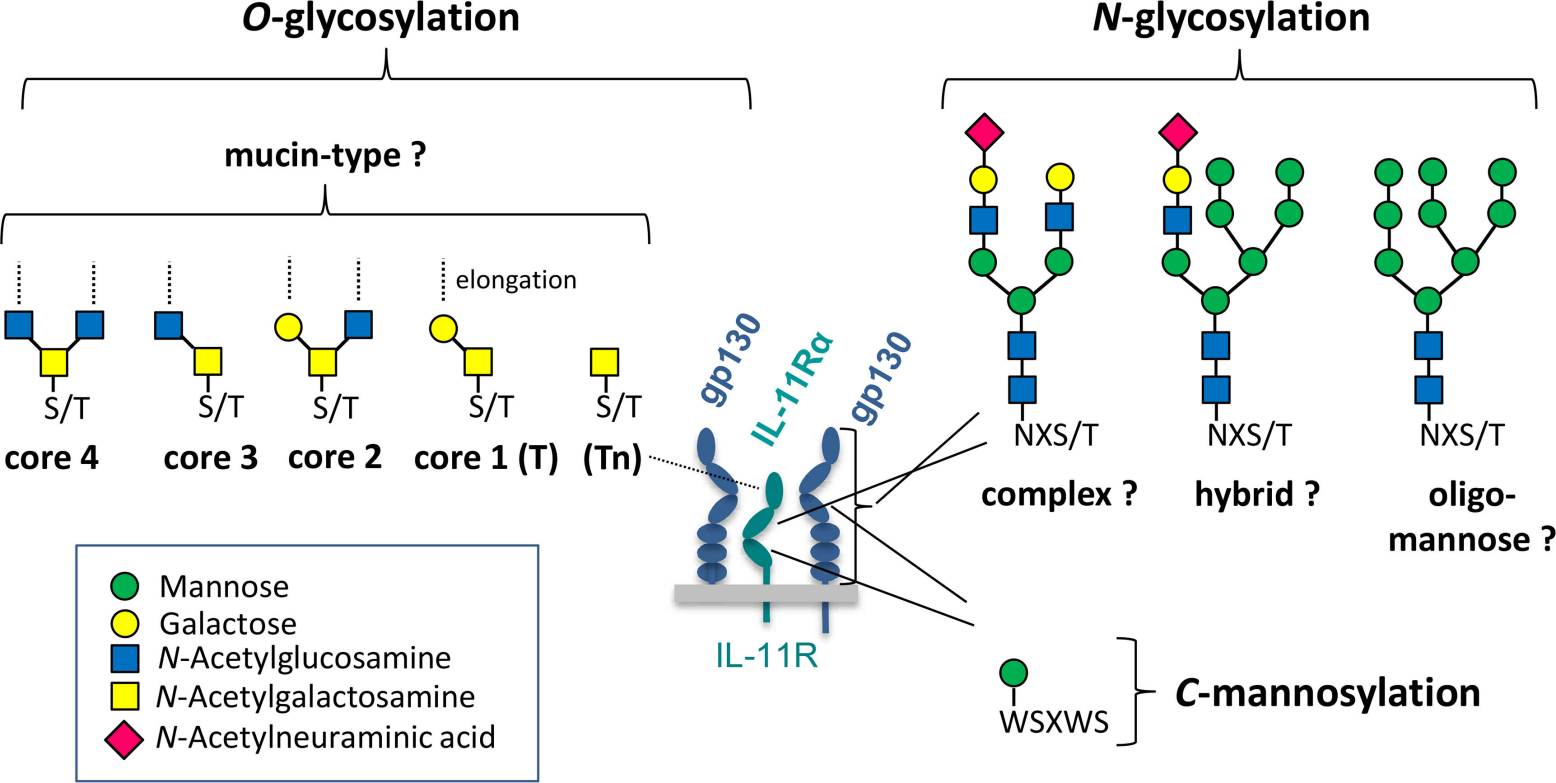
Proposed glycosylation of interleukin-6 family receptors. Receptors of the interleukin-6 family are known to be modified by *N*-glycosylation, *O*-glycosylation, and *C*-mannosylation. With the exception of *C*-mannosylation where the attachment of a single mannose has been experimentally proven for IL-11Rα, knowledge about specific structures of *N*- and *O*-glycans on these receptors is scarce. Characteristic structures of *N*- and *O*-glycans potentially attached to these proteins are depicted here as examples.

In this review, we summarize how the biology of IL-6 family cytokines (Figure 1) is regulated by glycosylation, a process in which carbohydrate chains are covalently linked to protein molecules (Figure 2). We describe the basic biology of how these post-translational modifications are attached to the protein via enzymes located in the endoplasmic reticulum (ER) and the Golgi apparatus. The attached carbohydrates can range from monosaccharides to complex structures and are divided into *N*-, *O*-, or *C*-linked glycosylation, depending on the amino acid residue they are attached to. Here, we summarize what is known about how the different kinds of glycosylation affect synthesis, receptor binding, signaling, and plasma half-life of the IL-6 family cytokines. Furthermore, we highlight the diverse functions of glycosylation on protein stability, transport to the cell surface, ligand binding, proteolysis, internalization, and recycling of the signaling and non-signaling receptors of the IL-6 family. Finally, we discuss how knowledge about glycosylation can be used for the design of novel therapeutics specifically targeting IL-6 family cytokines or their receptors. We describe what is known about the human proteins but will add information regarding cytokines and cytokine receptors from other species where available. As glycosylation is tightly connected to correct protein folding and errors in both processes are associated with a number of diseases, the principles discussed in this review are not limited to the IL-6 cytokine family but will be applicable to other protein families as well.

## Protein glycosylation

Glycosylation is the enzyme-catalyzed chemical modification of proteins with sugars (saccharides) and essentially affects physicochemical protein characteristics, for example, folding, conformation, solubility, and stability. Attachment of glycans to proteins further influences various biological protein functions comprising cell–cell- and cell–extracellular–matrix interaction, recognition, and signaling [9]. In this review, we will focus on *N*-, *O*-, and *C*-glycosylation, which are relevant in the context of cytokine biology. All these three types of protein glycosylation take place as co- or post-translational modifications, while the protein is passing through the secretory pathway via the ER and Golgi apparatus.

*N*-glycosylation is the most frequent form of protein glycosylation, and protein database analyses uncovered that about 70% of their entries contain a consensus motif of which a considerable proportion is likely to carry a glycan moiety [10]. This consensus motif consists of the sequon Asn-X-Ser/Thr (where X can be any amino acid except proline), where the oligosaccharides are attached to the nitrogen atom of asparagine via an *N*-glycosidic bond (Figure 2). The biosynthesis of *N*-glycans is initiated on a lipid-like carrier called dolichol phosphate at the cytoplasmic side of the ER. Subsequently, after flipping into the lumen of the ER, the *N*-glycan is further elongated and then transferred ‘en bloc’ to the nascent protein by the action of the oligosaccharyltransferase complex. Finally, the *N*-glycans are matured by trimming and reassembly, while the protein is passing through the Golgi apparatus. In a process comprising several glycosyltransferases and glycosidases, three main types of *N*-glycans are produced: high-mannose-, hybrid-, and complex-type glycans [10]. *N*-glycosylation of IL-6 family members has been widely described in literature to affect their biological function. In this review article, we will – to the best of our knowledge – for the first time comprehensively summarize and discuss the present literature on IL-6 family *N*-glycosylation.

Protein *O*-glycosylation occurs by the attachment of glycans to the hydroxyl group of serine or threonine residues building an *O*-glycosidic linkage between the sugar and the protein. In contrast to *N*-glycosylation, which always comprises the same core structure, *O*-glycans can be initiated with different saccharides. The protein characteristics, for example, specific domains, mostly determine the respective type of *O*-glycans that are attached. The most abundant form of *O*-glycosylation in mammals are mucin-type glycans, which are initiated by the attachment of an *N*-acetylgalactosamine to the carrier protein. This initial sugar (Tn antigen) can then be further modified by the addition of monosaccharides resulting in different core structures (e.g., core 1 known as T-antigen), which then build the basis for more complex *O*-glycans [11]. While it is widely accepted that IL-6 family members can carry *O*-glycans, knowledge about their specific structures and functions is scarce and we intend to elaborate on the significance of *O*-glycosylation for IL-6 family members in this review article (Figure 2).

A rather less intensively studied type of protein glycosylation is *C*-mannosylation, which involves the attachment of an α-mannose residue to the C2 atom of the indole ring of protein tryptophan residues [12]. This modification was first identified as a modification of RNase-2 in 1994 by Jan Hofsteenge [13]. *C*-mannosylation is catalyzed in the ER by enzymes designated as *C*-mannosyltransferases using the donor substrate dolichol phosphate mannose [14]. In mammals, four different homolog proteins exist (DPY19L1 to L4), and for just two of them (DPY19L1 and DPY19L3), *C*-mannosyltransferase activity has been proven so far [15]. The name relates to the *Caenorhabditis elegans* gene *dpy-19*, which belongs to a group of genes whose mutation causes a dumpy-like phenotype in the worms [16]. The consensus sequence for *C*-mannosylation is WXXW/C, where the first tryptophan becomes mannosylated. *C*-mannosylation has been identified on proteins containing thrombospondin type 1 domains [17], as well as on type-I cytokine receptors, for example, receptors of the IL-6-family like IL-11Rα [18] (Figure 2). It has been shown in several cases that *C*-mannosylation assists in protein folding and trafficking of proteins through the secretory pathway [12,18,19]. All cytokine-binding (IL-6Rα, IL-11Rα, CNTFR) and signaling (gp130, LIFR, OSMR, WSX-1, IL-31R) receptors of the IL-6 family contain a highly conserved WxxW motif (often referred to as WSXWS motif). Although it has not been experimentally proven so far, it appears highly likely that all the IL-6 family members that contain the consensus WXXW motif are target proteins for *C*-mannosylation. Based on our recent finding that a mutation affecting the consensus motif for *C*-mannosylation of the IL-11Rα caused a pathological phenotype in humans [18], we hypothesize a general importance of this modification in the biological function of IL receptors.

## The IL-6 family of cytokines

The IL-6 family of cytokines consists of nine secreted proteins that share the same three-dimensional protein fold, which consists of four alpha helices arranged in an up–up–down–down topology [4,5]. Furthermore, all cytokines of the family activate their target cells by using the signal-transducing β-receptor gp130 as either a homo- or a heterodimer [20] (Figure 1). The only exception is IL-31, which signals via a heterodimer of the oncostatin M (OSM) receptor (OSMR) and the IL-31 receptor (IL-31R)/ gp130-like receptor (GPL) [21]. The name-giving cytokine IL-6 [2] is not able to induce signaling directly but rather has to initially bind to a non-signaling α-receptor, the IL-6Rα (hereafter named IL-6R) [22]. Afterward, the complex of IL-6/IL-6R engages a homodimer of gp130 [23]. Similarly, IL-11 first binds to the non-signaling IL-11Rα (hereafter named IL-11R) and afterward activates intracellular signaling cascades via a gp130 homodimer [24,25].

Signaling of IL-6 and IL-11 via their membrane-bound α-receptors has been termed ‘classic signaling’ and is restricted to cells that express IL-6R and IL-11R, while gp130 is expressed ubiquitously [26]. In addition, soluble variants of both IL-6R and IL-11R exist (sIL-6R and sIL-11R, respectively), which bind IL-6 and IL-11 with the same affinity as their membrane-bound counterparts. The resulting IL-6/sIL-6R and IL-11/sIL-11R complexes can bind to and signal via gp130 homodimers, a process that is termed ‘trans-signaling’ and significantly expands the spectrum of cells that can be activated by the two cytokines, as it is not restricted by the expression pattern of the two α-receptors [27,28]. The sIL-6R is generated to a small extent by alternative splicing of the *IL6R* mRNA and predominantly by proteolytic cleavage of the membrane-bound IL-6R by the metalloprotease ADAM17 [29–32]. There is so far no evidence for alternative splicing of the *IL11RA* mRNA, and thus, proteolytic cleavage of the IL-11R by proteases like ADAM10 and RHBDL2 is the major mechanism for sIL-11R generation [33–36]. Furthermore, a third mode of IL-6 signaling termed ‘cluster signaling’ exists in which IL-6 via the membrane-bound IL-6R is presented from dendritic cells to cognate T cells, which are activated via own gp130 homodimers, a process that is required for the generation of pathogenic Th17 cells [37].

The other two cytokines that are not able to activate their β-receptors directly are ciliary neurotrophic factor (CNTF) and cardiotrophin-like cytokine factor 1 (CLCF1, which was previously named cardiotrophin-like cytokine (CLC)), both of which bind to the glycosyl–phosphatidyl–inositol (GPI)-anchored CNTF receptor (CNTFR) first before engaging a heterodimer of gp130 and leukemia inhibitory factor (LIF) receptor (LIFR) [38,39]. The same signal-transducing β-receptor complex is activated by cardiotrophin-1 (CT-1) [40], OSM [41] and LIF [42], which bind to their receptors directly and do not require an additional α-receptor. OSM can furthermore signal via a second receptor complex consisting of gp130 and OSMR [43]. The ninth family member is IL-27, a heterodimeric cytokine consisting of EBI3 (corresponding to a soluble α-receptor) and p28/IL-30 (the actual cytokine ligand) [44], which signals through a heterodimer of gp130 and the IL-27 receptor (IL-27R)/WSX-1 [45]. Formation of the individual signaling complexes results in the activation of several intracellular signaling cascades, which comprise the Janus kinase/signal transducer and activator of transcription (Jak/STAT), the phosphatidylinositol 3-kinase (PI3K), Src-YAP, and the extracellular regulated kinase (ERK/MAP) pathways [46–48].

All membrane-bound β-receptors also exist as soluble variants *in vivo* [49]. In contrast to the agonistic sIL-6R and sIL-11R, they compete with their membrane-bound counterparts for cytokine binding and thus act as inhibitors. While sgp130 has been used to generate therapeutics that specifically inhibit IL-6 and IL-11 trans-signaling [7,50–53], the other soluble β-receptors are largely unexplored and their therapeutic potential is not utilized.

## Influence of glycosylation on cytokine and cytokine receptor function

### Cytokines

#### IL-6 and viral IL-6

IL-6 is one of the most prominent pro-inflammatory cytokines and, thus, an important therapeutic target [3,54]. Antibodies that block either IL-6 or the IL-6R are in clinical use to treat inflammatory diseases like rheumatoid arthritis [7], and designer proteins like Olamkicept that target specifically IL-6 trans-signaling are currently investigated in clinical studies [26]. Furthermore, IL-6 has important physiological roles in T cell differentiation and tissue regeneration [3,54].

IL-6 has two potential *N*-glycosylation sites at N73 and N172 [1,55] and one *O*-glycosylation site at T170 [56] (Table 1). Early studies revealed that fibroblasts can produce IL-6 that is either *O*-glycosylated or both *N*- and *O*-glycosylated [65]. Interestingly, this appears to stem from differential cleavage of the signal peptide and thus different N-termini of IL-6: IL-6 proteins with A28 at the N-terminus are only *O*-glycosylated, whereas IL-6 proteins with V30 at the N-terminus carry both *N*- and *O*-glycosylation [66,67]. The underlying mechanism for this is not known, however.

**Table 1:** Glycosylation of IL-6 family cytokines.

Cytokine	*N*-glycosylation sites	Evidence for *N*-glycans	Function of *N*-glycans	Evidence for *O*-glycans
IL-6	N73, N172	Both sites confirmed [57,58]	Plasma half-life (N73), signaling (N73) under debate	Yes (T170) [58]
vIL-6	N78, N89	Both sites confirmed [59]	Binding to gp130 and signaling (N78), no role for N89 [59,60]	Unknown
IL-11	---	---	---	Unknown
CLCF1	N29	---	Unknown	Unknown
CNTF	---^2^	---	---	Unknown
CT-1	---^3^	---	---	Unknown
OSM	N100, N217	N100 (cryo-EM) [61]	Unknown; not involved in receptor binding [61]	Yes (T160, T162, and/or S165) [62]
LIF	N31, N56, N85, N95, N118, N127, N138	---	Unknown^5^	Unknown
p28/IL-30	---^4^	---	---	Indirect evidence [44]; T238 and S240 [63]
IL-31	N67, N100, N123	Indirect evidence [64]	Unknown	Unknown

*N*-glycosylation sites were predicted via NetNGlyc 1.0^1^ based on their Swiss-Prot ID. Information regarding evidence for their functionality, their biological function and evidence for *O*-glycans were obtained via literature review. See main text for further information.

1 https://services.healthtech.dtu.dk/services/NetNGlyc-1.0/.

2 CNTF has no signal peptide.

3 The murine CT-1 has a site at N164.

4 The murine p28/IL-30 has a site at N85.

5 In contrast with the human protein, glycosylation of rat and murine LIF has been investigated, which is described in detail in the main text.

*N*-glycosylation was later confirmed on IL-6 secreted from human blood mononuclear cells [57]. In this study, the authors were also able to determine the structures of the attached *N*-linked glycans. These included sialylated di- and tri-antennary complex-type and oligomannose-type structures and, most abundantly, a small tetrasaccharide with the sequence Manα6Manβ4GlcNAcβ4GlcNAc [57]. Several of these IL-6 glycoforms were later generated by semi-synthesis where the *N*-glycan was added to the N73 position [68]. In comparison with IL-6 expressed in *E. coli* and thus devoid of any *N*-linked glycosylation, all IL-6 variants were equally potent in inducing proliferation of Ba/F3-gp130-IL-6R cells in a dose-dependent manner, suggesting that here the different *N*-linked glycans had no influence on IL-6 signaling [68]. In contrast, a previous study found that IL-6 derived from mammalian cells had a stronger effect on the growth of B9 hybridoma cells compared to IL-6 derived from *E. coli* [69]. Glycosylated IL-6 was also more potent than unglycosylated IL-6 to elicit a rise of 1°C in body temperature in a rat model of pyrogenicity [70].

Different IL-6 glycoforms showed individual serum clearances depending on the structure of the *N*-glycan when injected into rats. Most IL-6 glycoforms were cleared slower than non-glycosylated IL-6, but their individual clearance was atypical, as the terminal α2,6-sialylated glycoforms of IL-6 cleared faster than the corresponding asialo IL-6 carrying *N*-glycans with terminal galactoses [68].

Recently, *N*-linked glycosylation of IL-6 at N73 was confirmed in IL-6 secreted from different lung cancer cells [58]. Furthermore, the authors showed that more than 99% of all IL-6 proteins carried a single *O*-glycan on T170. Interestingly, lung cancer cells also secreted an IL-6 variant with an *O*-, but no *N*-glycan, which they state derives from defective *N*-glycosylation due to a reduced *N*-glycosyltransferase gene expression. *N*-glycosylation-defective IL-6 induced shortened STAT3 activation compared with *N*-glycosylated IL-6 and altered the downstream signaling preference for the SRC-YAP-SOX2 axis, thereby inducing epithelial–mesenchymal transition and migration *in vitro* and contributing to metastasis *in vivo* [58]. It will be of great interest to determine whether such different IL-6 glycoforms also exist in other tumor entities or in inflammatory diseases and whether they actually have different functional properties in terms of strength and duration of the activated signaling cascades.

The functional relevance of *N*-glycosylation is much better established for viral IL-6 (vIL-6), a structural homologue of human IL-6 that is encoded by the human herpes virus 8 (HHV8)/Kaposi’s sarcoma-associated herpes virus [71]. Like IL-6, vIL-6 signals through a gp130 homodimer but does not require the IL-6R for doing so [72]. vIL-6 is *N*-glycosylated at N78 and N89 [59]. Interestingly, the glycosylation of vIL-6 at N89 enhanced binding to gp130 and thus the activation of the Jak/STAT signaling pathway [59]. Comparative experiments with IL-6 showed that neither *N*- nor *O*-glycosylation of IL-6 is necessary for binding to gp130 via the IL-6R nor the activation of Jak/STAT signaling and that unglycosylated and glycosylated IL-6 are equally potent to stimulate B cell proliferation [59]. A further study revealed that the glycans of vIL-6 attached at N78 are high mannose glycans, whereas complex glycans are attached at N89 [60]. Indeed, glycosylation at N89 affected the protein conformation of vIL-6, suggesting that these complex glycans contribute to a conformation of the cytokine that allows direct activation of gp130 without the need of an IL-6R [60].

Interestingly, the lack of cellular gp130 expression prevents vIL-6 secretion. Co-expression of vIL-6 inhibits the maturation of the *N*-glycans of gp130, suggesting intracellular binding of vIL-6 to gp130 and thus intracellular autocrine signaling from a pre-Golgi compartment [73]. In addition, vIL-6 interacts glycosylation-dependent with the ER-resident chaperon calnexin, which plays a role in ER localization of vIL-6 [74].

#### IL-11

IL-11 plays a role in a variety of chronic inflammatory diseases such as rheumatoid arthritis [75,76], as well as in the pathogenesis of fibrosis of various organs and tissues including the heart, lung, kidney, liver, and pancreas [77–79]. It is also involved in tumorigenesis and progression of several malignant neoplasms, including gastrointestinal [80,81] and breast cancers [82,83]. Several studies have already shown the therapeutic benefit of blocking IL-11 signaling, at least in mouse models [81,84]. As the role of IL-11 in the pathogenesis of various diseases becomes increasingly clear, the development of potent human IL-11 agonists and antagonists for treatment is essential. However, IL-11 is not only involved in pro-inflammatory processes, but it is also important for developmental processes such as bone homeostasis (reviewed in [85]) and female fertility [86–88], making it a challenging therapeutic target.

The amino acid sequence of IL-11 contains no *N*-linked glycosylation sites [89] (Table 1). Since glycosylation of proteins can improve their biological activity and stability [90], genetically modified variants of human IL-11 were generated and characterized [91]. For this purpose, *N*- and *O*-glycosylation sites were introduced into the cytokine sequence at the N-terminus (N), the first (M1), second (M2), or third (M3) loop connecting the alpha helices, or at the C-terminus (C). Expression of the IL-11 variants in various mammalian cell lines and analyses of their expression via Western blot showed that N, M1, and M3 variants had a higher level of glycosylation than M2 or C variants [91].

N-terminally modified variants of IL-11 appear to be potent agonists, whereas the introduction of glycosylation sites in the M1 loop resulted in IL-11 variants that can act as antagonists. It remains unclear whether the glycans themselves modify binding affinity to the receptor or whether this is due to a structural change. C-terminally modified IL-11 variants do not appear to be glycosylated or only slightly glycosylated and function similarly to wildtype. C-terminal glycosylation is, therefore, not a candidate modification for more potent IL-11 [91].

#### CLCF-1

CLCF-1 acts within the immune system due to the stimulation of B cells and fulfills certain neurotrophic functions, including neural differentiation and survival [92]. CLCF-1 has one predicted *N*-linked glycosylation site (Table 1), but it has not yet been investigated whether it is actually used and it is important for the biological functions of CLCF-1.

#### CNTF

CNTF is a special member of the IL-6 family as its immature form does not contain a classical signal peptide and it is instead believed to be released from neuronal cells after injury [93,94]. Therefore, CNTF does not travel along the secretory pathway and does not meet the enzymes required for the attachment of glycans within the ER and the Golgi apparatus (see section 2). Thus, CNTF should not be modified by glycosylation, and its biological functions are, therefore, independent of such post-translational modifications (Table 1).

#### CT-1

The amino acid sequence of human CT-1 does not contain a sequon that would allow *N*-linked glycosylation (Table 1). However, the mouse CT-1 contains a potential *N*-linked glycosylation site [95], and recombinant murine CT-1 has an apparent molecular weight of about 30 kDa, which is indicative of a glycoprotein, as the pure polypeptide would only generate a 22-kDa protein [96]. Further detailed studies, however, especially in terms of a functional relevance of the *N*-linked glycans, have not been performed to date.

#### OSM

OSM has diverse roles in various processes such as bone remodeling, wound healing, liver regeneration, and inflammatory bowel disease [97,98]. OSM has two predicted sites for *N*-glycosylation (Table 1), but these are sparsely analyzed so far. Recent structures of both OSM signaling complexes provided evidence for *N*-linked glycan densities at N100 of human OSM [61]. It has further been shown that a glycosylated form of OSM interacts less stably with the extracellular matrix (ECM) than non-glycosylated OSM, letting the authors speculate that the *N*-linked glycans interfere with the binding sites on the ECM [99]. In a different study, it was shown that OSM exists in two different glycoforms and that these have different functions in spermatogenesis [100]. Although not thoroughly investigated, a mutant of human OSM in which both sequons are altered so that no *N*-glycan can be attached was biologically active [62]. In the same study, the presence of up to nine attached hexose units was shown, which indicates the presence of *O*-glycans [62]. However, no functional experiments were carried out in which glycosylated and unglycosylated OSM were compared.

#### LIF

LIF was first described as a cytokine that was able to inhibit the proliferation of myeloid leukemia cells, but it is nowadays clear that it has important roles in several physiological and pathological conditions, including infection and inflammatory bowel disease [101]. With seven potential *N*-glycosylation sites, human LIF is the cytokine of the IL-6 family with the most possible post-translational modifications (Table 1). Although it is normally produced as a glycoprotein, a non-glycosylated LIF is still biologically active [102]. While the impact of the individual N-glycans of human LIF still remains unknown, the function of the six individual *N*-glycans of rat LIF has been investigated in detail: the authors generated six *N*-glycosylation-deficient LIF mutants by replacing each asparagine residue with a glutamine residue and investigated their biological activity using two different cell lines. LIF-N34Q was about three times more potent than the wildtype LIF, whereas LIF-N63Q was about 2.5 times less potent than wildtype LIF, suggesting diverging roles of these two *N*-glycosylation sites. However, these results were only obtained with one cell line, and in contrast, all LIF mutants showed the same biological activity with the second cell line tested [103]. It is therefore questionable whether these results reveal a general mechanism how *N*-linked glycans influence the formation of or signaling via the LIFR/gp130 signaling complexes or whether they are rather a specific property of a single cell line. In line with the data from human LIF, a rat LIF devoid of all *N*-glycans was still biologically active [104]. Although murine LIF is also heavily glycosylated [105,106], similar mechanistic experiments have not been performed. It has been speculated that the glycosylation of LIF influences the ratio between apical and basolateral secretion [107], as it has been shown for erythropoietin [108], but this has not been further evaluated.

#### p28/IL-30 (IL-27)

IL-27 has important roles in inflammation, infection, cancer development, and regulation and differentiation of immune cells [109]. Murine p28/IL-30 can be secreted from cells without EBI3, whereas human p28 required co-expression of EBI3 for its secretion [44,110]. Murine p28/IL-30 has one sequon for *N*-linked glycosylation at N85, which is absent in human IL-27 (Table 1, [110]). Instead, several *O*-glycosylation sites can be predicted from the amino-acid sequence of the cytokine, and the expression of human p28 in HEK293T cells revealed three bands at apparent molecular weight between 25 and 29 kDa in the cell lysate, probably reflecting different *O*-linked glycosylation states [44]. Indeed, a recent study confirmed *O*-glycosylation at T238 and S240 but revealed that a complex of EBI3 with the *O*-glycan-deficient p28 was fully biologically active, suggesting that *O*-linked glycosylation of p28 was dispensable for signaling [63].

#### IL-31

IL-31 is produced by Th2 cells, mast cells, monocytes and macrophages, and dendritic cells [43] and plays important roles in different human diseases like atopic dermatitis [21] and airway hypersensitivity [111]. IL-31 has three potential *N*-glycosylation sites (Table 1), which appear to be used, as several native IL-31 isoforms ranging from 24 to 33 kDa were observed in the supernatant of a human Th2 cell line [64]. However, neither the chemical nature of the glycans (microheterogeneity), the positions to which of the three asparagine residues the glycans are linked (macroheterogeneity) nor their biological functions have been investigated to date. Notably, recombinant IL-31 produced in bacteria was biologically active, ruling out a major role for *N*-linked glycans in the signal transduction of IL-31 via OSMR/IL-31R [112].

### Cytokine alpha receptors

#### IL-6R

The ectodomain of the IL-6R consists of an immunoglobulin (Ig)-like domain (‘D1’) and two fibronectin-type-III domains (‘D2’ and ‘D3’), which contain the cytokine-binding module. It is connected to the transmembrane and the intracellular region via a linker consisting of 52 amino acid residues, the so-called stalk region [113]. The stalk acts as a spacer that keeps the ectodomain in a certain distance from the plasma membrane in order to allow complex formation with the two gp130 molecules [114].

Early *in vitro* studies had identified some asparagine residues of the IL-6R that were used for *N*-linked glycosylation [115,116], but neither *O*-linked glycosylation of the IL-6R was analyzed nor a systematic and mechanistic investigation into glycosylation of the IL-6R was performed until recently. Here, the occupancy of all *O*- and *N*-glycosylation sites on a sIL-6R form isolated from human serum was determined using liquid chromatography–mass spectrometry [32] (Table 2). Functional analyses using different genetically engineered Ba/F3-gp130 cell lines revealed that *N*- and *O*-glycosylation were dispensable for signaling of the IL-6R. However, proteolysis by the metalloproteases ADAM17 and ADAM10 was orchestrated by an *N*- and *O*-glycosylated sequon in the stalk region near the cleavage site and an *N*-glycan exosite in domain D1, and proteolysis of an IL-6R completely devoid of glycans was significantly impaired, revealing that glycosylation is an important regulator for sIL-6R generation [32]. Whether *N*-glycans are structurally different from IL-6R proteins derived from different cell types or whether the structures of glycans differ between healthy individuals and patients has not been investigated so far.

**Table 2:** Glycosylation of IL-6 family cytokine receptors.

Receptor	*N*-glycosylation sites	Evidence for *N*-glycans	Function of *N*-glycans	Evidence for *O*-glycans
IL-6R	N55, N93, N221, N245, N350	All confirmed [32]	N55 and N350 regulate proteolysis [32]	T352, regulates proteolysis [32]
IL-11R	N127, N194	Both (mutagenesis) [117], N194 (mass spectrometry) [18]	Transport to the cell surface (N194), regulation of proteolysis (N127/N194) [117]	Yes [117,118]
CNTFR	N60, N70, N142, N190	All confirmed [119]	Unknown	Unknown
EBI3	N55, N105	All confirmed [63]	Essential for secretion and signaling [63]	Unknown
gp130	N43, N83, N131, N157, N227, N246^1^, N379, N383, N390^1^, N553, N564	N43, N83, N131, N157, N227, N379, N383, N553, N564 [120,121]; N131, N157, N227 (cryo-EM) [61]	*N*-glycosylation is required for the stability, but not for signal transduction [121]	Unknown
LIFR	N64, N85, N131, N143, N191, N243, N303, N390, N407, N426, N445, N481, N489, N572, N652, N663, N680, N729, N787	N303, N407, and N426 (cryo-EM) [61]	PNGaseF reduces LIF-induced STAT3 signaling [122]	Unknown
OSMR	N42, N84, N131, N163, N176, N221, N307, N326, N345, N361, N380, N422, N446, N491, N509, N580	---^2^	Unknown	Unknown
WSX-1	N51, N76, N302, N311, N374, N382, N467	---	Unknown	Unknown
IL-31R	N27, N37, N67, N71, N93, N166, N183, N187, N277, N283, N380, N395, N455, N473	---	Unknown	Unknown

*N*-glycosylation sites were predicted via NetNGlyc 1.0 based on their Swiss-Prot ID. Predicted sites not located in the extracellular domain of the proteins have been discarded. Information regarding evidence for their functionality, their biological function, and evidence for *O*-glycans were obtained via literature review. See main text for further information.

1 *N*-glycosylation site is not functional [120].

2 N162, N239, N271, M304, and N323 of the murine OSMR have recently been confirmed as functional *N*-glycosylation sites by cryo-EM [61].

Thus, glycosylation of the IL-6 receptor is important for the regulation of limited proteolysis and thus the initiation of IL-6 trans-signaling, but it is also involved in folding, intracellular trafficking, stability, and cell surface expression.

#### IL-11R

The IL-11R has the same topology as the IL-6R [113,123]. The D3 domain carries the typical WSXWS motif found in many type-I cytokine receptors, which is involved in folding, ligand binding, and intracellular trafficking [124–126]. The amino acid sequence of the IL-11R predicts a protein with a molecular weight of 30 kDa. In 1997, Curtis et al. showed via experiments using *N*-glycanase F, an enzyme that cleaves *N*-linked glycans, that the murine-soluble IL-11R has two functional *N*-glycosylation sequons and may carry additional *O*- or *C*-glycans (Table 2, [118]). A later study confirmed two *N*-glycosylation sites within the D2 domain of the human IL-11R at N127 and N194, and at least one *O*-glycan [117]. The mutation of the *N*-glycan sites leads to a reduced cell surface amount of the IL-11R and is, therefore, relevant for intracellular transport, stability, and surface expression of the IL-11R. Here, the *N*-glycan site N194 seems to be more relevant than N127, as the IL-11R-N194Q was retained in the ER, which was not the case for N127. *N*-glycosylation was dispensable for IL-11-induced signaling but required for efficient IL-11R proteolysis by the metalloprotease ADAM10 [117].

Several coding mutations in the *IL11RA* gene, which encodes the IL-11R, are associated with craniosynostosis, a condition in which the cranial sutures close prematurely, resulting in craniofacial abnormalities and other symptoms. The mutations are distributed over the entire ectodomain of the receptor (reviewed in [85]). As described previously in Section 2, the glycosylation status of a protein changes as it travels along the secretory pathway, which can be used to monitor maturation and transport of the protein. The analysis of lysates of cells expressing different IL-11R patient variants by Western blot revealed that the band of the IL-11R with the highest apparent molecular weight, which represents the fully matured IL-11R at the cell surface, was completely absent in most patient mutations, indicating retention in the ER, incomplete glycosylation, and a lack of functional IL-11R at the cell surface [18,127,128]. However, other IL-11R mutations result in at least small amounts of IL-11R on the cell surface, which is sufficient for proper IL-11 signaling [129].

A key feature of cytokine receptors of the IL-6 family is the presence of a WSXWS motif, which is a site for *C*-mannosylation [15,19]. In the IL-11R, the WSXWS motif is located in domain D3 and part of a so-called arginine–tryptophan zipper [127]. Analysis of public proteomic data confirmed that the IL-11R is indeed *C*-mannosylated at the WSXWS motif and fully *N*-glycosylated at N194, while it is only partially *N*-glycosylated at N127 [18]. Some craniosynostosis-associated mutations affect this zipper, most likely causing misfolding and therefore ER retention. Additionally, the patient mutation p.T306_S308dup results in a duplication in the WSXWS motif [18]. All these variants showed incomplete maturation, lack of surface expression, and thus abrogation of downstream signaling of the IL-11R. This highlights the importance of an intact arginine–tryptophan zipper and the WSXWS motif for IL-11R maturation and suggests that mutations disrupting these structural motifs cause intracellular retention. Whether this is due to misfolding or the lack of *C*-mannosylation at the WSXWS motif or both remains currently unclear. The importance of *C*-mannosylation has also been demonstrated for other transmembrane proteins, suggesting a common role for this modification [19,130].

In conclusion, glycosylation of the IL-11 receptor is important for folding, intracellular trafficking, stability, and cell surface expression. The WSXWS motif and the arginine–tryptophan zipper appear to be of particular importance for the IL-11R, as mutations in these motifs lead to retention in the ER and thus to abrogation of IL-11-dependent signaling.

#### CNTFR

The CNTFR is unique in the IL-6 cytokine family since it is linked to the plasma membrane via a GPI-anchor and therefore does not contain a transmembrane region and no intracellular region [39]. Mechanistically, however, these different biochemical properties have no influence, as the CNTFR serves as the α-receptor for CNTF and thus has the same principle function as the IL-6R and the IL-11R – namely that only the CNTF/CNTFR dimer can induce the formation of the heterodimer of the signal-transducing β-receptors gp130 and LIFR.

The CNTFR has four putative sites for *N*-linked glycosylation, which are all used (Table 2, [119]). *In vivo*, rat CNTFR displays different glycosylation patterns in different organs, with brain and stomach being the organs that have CNTFR with the highest apparent molecular weight. Importantly, after treatment with PNGaseF, CNTFR gel bands merged into a single band with a distinct molecular weight [131]. Functional studies regarding the individual *N*-linked glycans have not been performed yet, and a mechanistic explanation for the organ-specific glycosylation pattern is not known so far.

#### EBI3

EBI3 is part of the heterodimeric cytokine IL-27 [44]. Whereas p28/IL-30 represents the cytokine subunit, EBI3 functions as the cytokine receptor. Of note, EBI3 is the only α-receptor of the IL-6 family that does not exist as a membrane-bound receptor but is only expressed as a soluble protein. Importantly, EBI3 can also be secreted without p28 [44]. EBI3 contains two sites for *N*-linked glycosylation [132] (Table 2). Treatment of EBI3 with N-glycanase resulted in a shift in the apparent molecular mass from 33 to 28 kDa, suggesting that both sequons are actually used [132]. This initial observation was recently confirmed [63,133].

Interestingly, glycosylation of EBI3 is essential for secretion without a binding partner, as the EBI3-N55Q/N105Q double mutant was not secreted alone. However, both EBI3 wildtype and the double mutant supported the secretion of p28 and the *O*-glycan-deficient mutant p28-T238A/S240A, revealing that the glycosylation of EBI3 is required for its secretion alone and that the secretion of assembled IL-27 is independent of *N*-linked glycosylation of EBI3 [63]. Furthermore, EBI3 without *N*-linked glycosylation showed decreased biological activity compared with IL-27 wildtype [63].

### Cytokine beta receptors

#### gp130

gp130 is the signal-transducing β-receptor for all but one cytokine of the IL-6 family, and it is not surprising that it is the best-studied β-receptor in terms of its glycosylation. It contains 11 potential *N*-glycosylation sites, of which nine are actually used (Table 2, [120]). The first structure of an extracellular gp130 cytokine receptor signaling complex was generated from proteins expressed in cells treated with tunicamycin, an inhibitor of *N*-linked glycosylation [134]. A recent structure of the IL-27 complex contained *N*-linked glycans in the gp130 protein, but these were added to alphafold models and were not derived experimentally [135]. However, a recent structure of the OSM signaling complex provided evidence for *N*-linked glycan densities on N131, N157, and N227 of human gp130, although they are not directly involved in interactions [61].

Cellular experiments with tunicamycin have resulted in divergent and rather inconclusive results regarding the functional consequences of *N*-glycosylation of gp130 [136,137]. A thorough functional study showed that mutation of all nine functional *N*-glycosylation sites by replacing the asparagine residues with glutamine results in a gp130 variant whose majority is not transported to the cell surface, but rather degraded via the proteasome. However, the small fraction that reaches the cell surface is biologically active and induces signaling when stimulated with IL-6/sIL-6R. The authors concluded that *N*-linked glycosylation is required for the stability, but not the signal-transducing function of gp130 [121]. This was further substantiated by a study in which cytokine signaling in cells from patients with mutations in phosphoglucomutase 3 (PGM3) was analyzed [138]. PGM3 is an enzyme required for the synthesis of N-acetylglucosamine, a precursor necessary for *N*-linked glycosylation. Cells from these patients responded much weaker to the stimulation with IL-6 and IL-27 compared to control cells because the reduced function of PGM3 resulted in a lack of fully glycosylated gp130 proteins and therefore reduced gp130 cell surface levels [138]. Signaling of cytokines which do not require gp130 for signaling like IL-10 and IL-21 was not affected. Consistent with this, the co-expression of vIL-6 prevents the complete maturation of gp130 *N*-glycans due to interaction and thus retention within intracellular compartments [73].

#### LIFR

The human LIFR contains 20 potential *N*-glycosylation sites, of which 19 are located within the extracellular domain (Table 2, [139]). LIFR was shown to be expressed in different tissues of the rat, including epididymal fat, brain, lung, skeletal muscle, and testes [131]. PNGaseF treatment of lysates of the different tissues resulted in a marked difference of the apparent molecular weight, indicating *N*-linked glycosylation [131]. Whether all known sites for *N*-linked glycans are functional is not known, but the recent structure of the OSM signaling complex provides evidence for glycosylation at N303, N407, and N426 [61].

*N*-glycosylation of endometrial cells contributed to the successful implantation of the mouse embryo, and removal of *N*-glycans via PNGaseF treatment or inhibition of *N*-glycosylation by tunicamycin of human endometrial cell lines impaired their receptive ability to human trophoblastic JAR cells [122]. Mechanistically, the *N*-glycosylation of the LIFR reduces the receptive potentials of endometrial cells via regulation of LIF-induced STAT3 signaling [122]. Recently, aberrant glycosylation of soluble LIFR in the sera from pancreatic cancer patients was reported, which might serve as a biomarker [140].

#### OSMR

The amino acid sequence of the human OSMR contains 16 potential *N*-linked glycosylation sites in its extracellular domain (Table 2). No functional data for the human OSMR have been published so far, and only recently the first study reported that N162, N239, N271, M304, and N323 of the murine OSMR indeed carry *N*-glycans. However, none of these glycans were directly involved in the binding of OSM [61].

#### WSX-1

The amino acid sequence of human WSX-1 contains seven potential *N*-linked glycosylation sites, and five of these are conserved in the murine WSX-1 (Table 2, [141]). No functional analyses have been performed to date, but a recent structure of IL-27 reported ‘enzymatic shaving of *N*-linked glycosylation’ before obtaining protein crystals, suggesting that at least some of the *N*-glycosylation sites are indeed functional [142]. Another structure of the IL-27 complex contained *N*-linked glycans on WSX-1, but these were not derived experimentally but added later to alphafold models [135].

#### IL-31R/GPL

The IL-31R (also known as gp130-like receptor or GPL [143]) has 14 predicted *N*-glycosylation sites (Table 2). No functional studies have been performed to date with this cytokine receptor.

## Therapeutic implications

Engineering of cytokines and soluble cytokine receptors holds great promise for the development of novel therapeutics [144–146]. Importantly, 80% of cytokines relevant for therapeutic purposes are glycoproteins [147]. Modifying attachment sites for glycans or engineering of the specific carbohydrate chains that are added to the protein opens up an intriguing and yet largely unexplored field of biomedical protein engineering. Importantly, current biotherapeutics are rather heterogenous in terms of their glycosylation, which can have a profound influence on stability, plasma half-life, and, in the end, the efficacy of the therapeutic [148].

At least for the IL-6 cytokine family, glycosylation appears not to be essential for the initiation of signal transduction. With the exception of some early reports on IL-6 itself, which have not been reproduced later, and one study on LIF, which only showed altered biological activity depending on the *N*-glycosylation in a single cell line, *N*- and *O*-linked glycans on both the cytokines and their receptors are dispensable for binding to each other. Several studies have shown that unglycosylated cytokines and receptors are fully biologically active, suggesting that the functional role of the glycans might be something else, ensuring protein stability and allowing intracellular traveling along the secretory pathway. This is important to know because monoclonal antibodies that bind to and thereby block cytokines and their receptors can be developed against the unglycosylated proteins, and glycosylation does not have to be taken into account. Furthermore, there are multiple examples that cytokines are present as different glycoforms with varying *N*-, *O*-, or *C*-glycosylation, depending on the cell type that produces the cytokine or pathological conditions [147]. Thus, when injected into a patient, an antibody faces a heterogeneous mixture of differentially glycosylated and non-glycosylated variants of its target protein, and it is important to ensure that the neutralizing capacity of the antibody is independent of the glycosylation of the target.

Recently, the first reports of aberrant glycosylation pattern of cytokines and soluble cytokine receptors in patients with pancreatic and lung cancer have been published [58,140]. Besides the above-mentioned bioengineering approaches, using glycosylation as a biomarker to detect or monitor diseases in blood or urine samples of patients might be a promising and non-invasive avenue to use glycosylation for the benefit of the patients. Understanding the underlying biological mechanisms that lead to such altered glycosylation patterns will keep researchers busy for the next decades.

## Abbreviations

CLCF 1: cardiotrophin-like cytokine factor 1
CNTF: ciliary neurotrophic factor
CNTFR: CNTF receptor
ER: endoplasmic reticulum
IL-6: interleukin-6
Jak/STAT: Janus kinase/signal transducer and activator of transcription
LIF: leukemia inhibitory factor
LIFR: LIF receptor
OSMR: oncostatin M receptor
